# Controlling Nutritional Status (CONUT) Score as a New Indicator of Prognosis in Patients With Hilar Cholangiocarcinoma Is Superior to NLR and PNI: A Single-Center Retrospective Study

**DOI:** 10.3389/fonc.2020.593452

**Published:** 2021-01-11

**Authors:** Ankang Wang, Zhenxing He, Peng Cong, Yueyu Qu, Tao Hu, Yu Cai, Bo Sun, Hao Chen, Wenguang Fu, Yong Peng

**Affiliations:** ^1^ Department of General Surgery, Nanchong Central Hospital, The Second Clinical College of North Sichuan Medical College, Nanchong, China; ^2^ Department of Hepatobiliary Surgery, The Affiliated Hospital of Southwest Medical University, Luzhou, China

**Keywords:** hilar cholangiocarcinoma, controlling nutritional status score, neutrophil lymphocyte ratio, prognostic nutritional index, prognosis

## Abstract

**Background:**

Currently, many nutritional indicators, including controlling nutritional status score (CONUT), can be used to assess a patient’s nutritional status and have been reported as reliable predictors of multiple malignancies. However, the value of CONUT score in predicting postoperative outcomes in patients with hilar cholangiocarcinoma has not been explored. In this study, its predictive value will be discussed and compared with the known predictors the neutrophil lymphocyte ratio (NLR) and prognostic nutritional index (PNI).

**Methods:**

Preoperative CONUT scores, PNI and NLR levels of 94 Hilar cholangiocarcinoma (HCCA) patients who underwent radical-intent resection of hepatobiliary surgery in our hospital from March 2010 to April 2019 were retrospectively collected and analyzed. They were grouped according to their optimal cutoff value and the prognostic effects of patients in each group were compared respectively.

**Results:**

CONUT^high^ was more frequent in patients with Clavien–Dindo classification of ≥IIIa (P = 0.008) and Bile leakage presence (P = 0.011). Kaplan-Meier curves analyzing the relationship between CONUT, PNI, and NLR values and HCCA patient survival (including total survival (OS) and recurrence-free survival (RFS) showed significant differences between groups (P <0.001). Meanwhile, multi-factor analysis found that Degree of cure, PNI, NLR, and preoperative CONUT score were independent prognostic factors for OS and RFS. The predictive power of CONUT score was higher than that of NLR and PNI based on time-dependent receiver operating Characteristic (ROC) analysis and the net reclassification index (NRI) and integrated discriminatory index (IDI) values (P < 0.05).

**Conclusion:**

CONUT score may be of some clinical reference value in evaluating postoperative prognosis of HCCA patients.

## Introduction

Hilar cholangiocarcinoma (HCCA), also known as Klatskin tumor, is a malignant tumor located at the bifurcation of the hepatic duct and originating from the bile duct epithelium (1, 2). Because the disease is usually advanced and no effective adjuvant therapy is available, this type of tumor is considered to have a worse prognosis than intrahepatic or distal cholangiocarcinoma (3, 4). However, despite the rapid development of surgical techniques today, the 5-year OS rate of HCCA patients is only 10–44% (5–7). An estimated 17–46% of surgical patients are diagnosed with malnutrition at the time of admission (8–10), which can lead to delayed wound healing and dysfunction of immune cells such as neutrophils, macrophages and lymphocytes (11).

A number of studies have confirmed that malnutrition dramatically increase the occurrence of postoperative complications in cancer patients, negatively affects the anticancer efficacy, length of hospital stay and quality of life of cancer patients, and also speeds up the progress of cancer, leading to poor survival (11–14). Since nutritional status may be an important factor affecting the degree of liver regeneration, and preoperative malnutrition is highly correlated with the incidence of postoperative liver failure and postoperative death, this phenomenon is particularly evident in the field of liver surgery (15–17). Based on the above findings, several nutrition-based scores have been identified in recent years as possible prognostic markers for various cancers, and the details of these scores are readily available from peripheral blood samples (18, 19). Among them, the neutrophil lymphocyte ratio (NLR) and prognostic nutritional index (PNI) were considered to be independent prognostic factors that affect the survival of many malignancies (20–23), and the Controlled Nutritional Status (CONUT) score, as an immune nutritional status scoring system that emerged in 2005, also took serum cholesterol into account when assessing the immune nutritional status of patients compared to PNI and NLR (24, 25). The CONUT score, which is comprised of the serum values of albumin (ALB), total lymphocyte count (TLC), and total cholesterol, is considered to be a prognostic factor for postoperative prognosis of malignant tumors such as carcinoma of colon, non-small cell lung cancer, liver cancer and pancreatic ductal adenocarcinoma (26–29), and has also been reported to be an important prognostic factor for survival in non-resectable HCCA (30).

However, there are few studies on the effect of nutrition score on the prognosis of patients with hilar cholangiocarcinoma. This study aims to assess the role of CONUT score in evaluating the prognosis of patients with this particular type of tumor and to compare its efficacy with PNI and NLR indicators.

## Materials and Methods

### General Information

The clinicopathological data of patients with HCCA who underwent radical-intent resection in the Affiliated Hospital of Southwest Medical University from March 2010 and April 2019 were retrospectively analyzed.

Case inclusion criteria (1): Radical-intent resection was performed, and postoperative pathology confirmed HCCA (2); No adjuvant therapy was given before the operation; (3) No systemic metastatic lesions were found before surgery; (4) No history of other malignant tumors; (5) Complete clinical and follow-up data. Case exclusion criteria: (1) Non-primary HCCA; (2) Other biliary tract diseases occurred before operation; (3) Complications of cardiovascular and cerebrovascular diseases, kidney diseases or blood system diseases before operation; (4) Steroid use within 15 days prior to surgery or other known autoimmune diseases; (5) Recent history of blood transfusion or preoperative immunoenhancement treatment; (6) Death occurred within 30 days after surgery; (7) Only palliative internal drainage or palliative R2 resection was performed.

### Investigational Variables

We collected all clinicopathological data through the electronic medical record system. The data included gender, age, serum carcinoembryonic antigen (CEA), CA199 levels and TBIL, and other pathological information such as histological type, tumor size, and Bismuth-Corlette classification.

The invasion and metastasis of hepatic parenchyma, lymph node, hepatic artery, portal vein and AJCC stage were evaluated. Blood samples was collected one week before surgery to assess total peripheral blood lymphocyte count, total cholesterol level, serum albumin, aminotransferase (AST), alanine aminotransferase (ALT), platelet (PLT), neutrophils and other biochemical and coagulation indicators. The tumor size was determined by the maximum tumor diameter and tumor staging was performed according to the AJCC Cancer Staging Manual (8th edition) (31).

The CONUT, NLR, and PNI values were calculated according to the blood routine examination and biochemical results. Complications were assessed using the incidence of bile leakage (32) and Clavien-Dindo classification (33). The CONUT score is calculated using the method shown in **Table 1**.

**Table 1:** Scoring system for the controlling nutritional status (CONUT).

Degree of undernutrition	CONUT score	Serum albumin (g/dl)	Total lymphocyte (/mm3)	Total cholesterol (mg/dl)
Normal	0–1	≥3.50 (0)	≥1,600 (0)	≥180 (0)
Mild	2–4	3.00–3.49 (2)	1,200–1,599 (1)	140–179 (1)
Moderate	5–8	2.50–2.99 (4)	800–1,199(2)	100–139 (2)
Severe	9–12	<2.50 (6)	<800 (3)	<100 (3)

CONUT score =Serum albumin score + total lymphocyte score + total cholesterol score.

All subjects in this study have signed informed consent forms and have been examined and approved by the Ethics Committee of the Affiliated Hospital of Southwest Medical University.

### Follow-Up Situation

Regular postoperative follow-up was conducted for the included patients. Follow-up methods include outpatient reexamination and telephone follow-up. Patients were told to return to the hospital for a review every 3 months for the first year and every 6 months thereafter.

**Figure 1:**
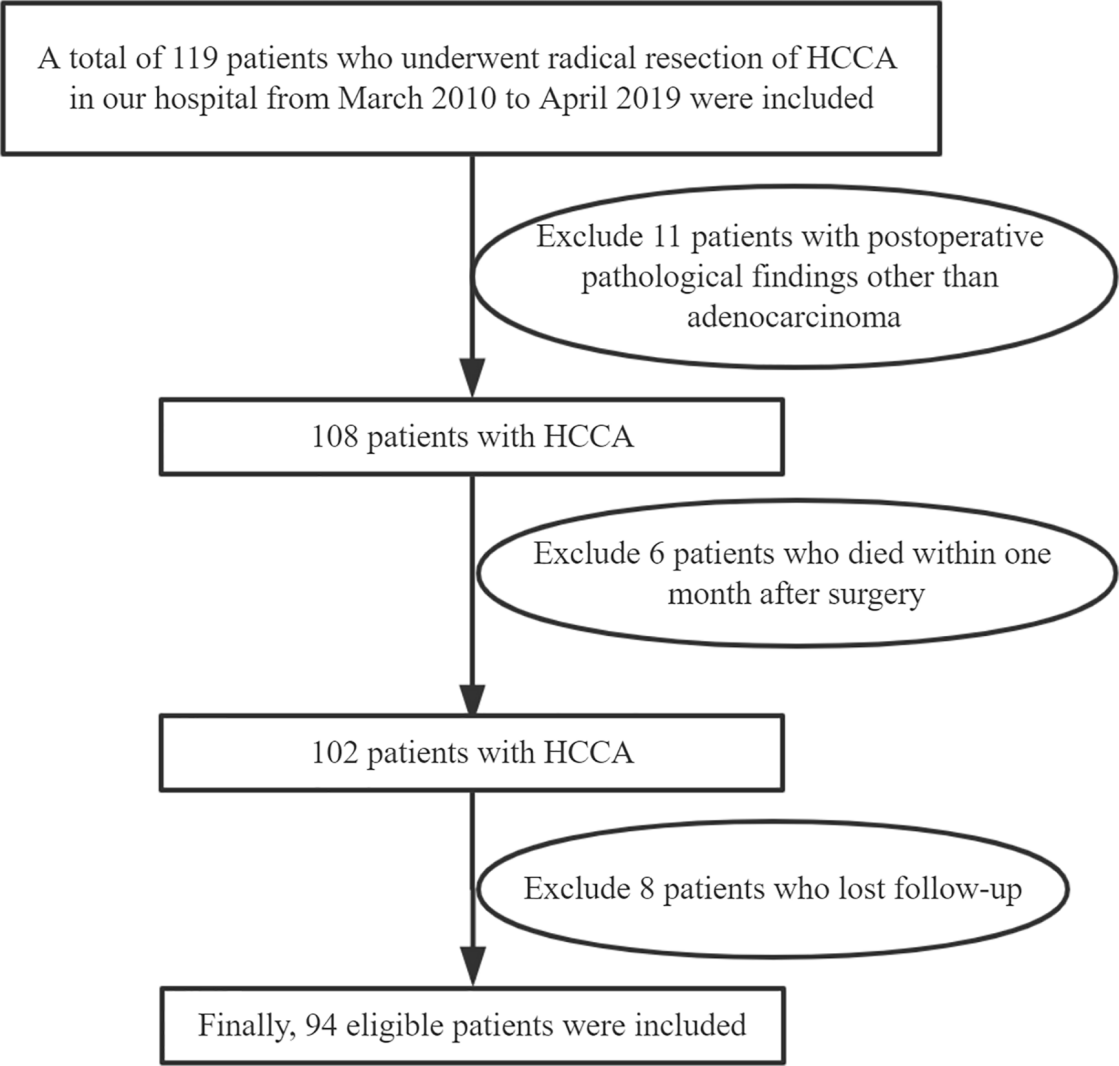
Enrollment and outcomes. HCCA, Hilar cholangiocarcinoma.

The items reviewed included CA19-9, CEA, liver function, blood routine, and abdominal ultrasound. If there are unexplained symptoms or suspicious lesions, magnetic resonance imaging (MRI), contrast-enhanced computed tomography (CT), positron emission tomography (PET), and other examinations can be used to aid diagnosis. Survival time is expressed by recurrence-free survival (RFS) and overall survival (OS). Patient enrollment and follow-up are shown in **Figure 1**, and 94 patients were eventually enrolled in this study, and the follow-up deadline was April 2019.

### Definition of CONUT, NLR, and PNI Values

The optimal cutoff values of preoperative CONUT, NLR and PNI were obtained, which were 3, 3.6, and 43.7 respectively, and they were reclassified into the high group and the low group. As shown below: CONUT^low^(<3; n = 63) and CONUT^high^ (≥3; n = 31), PNI^low^ (<43.7; n = 44) and PNI^high^ (≥43.7;n = 50), NLR ^low^ (<3.6; n = 43), NLR ^high^ (≥3.6;n = 51). In addition, the optimal cutoff values of TBIL (65.9 μmol/L), CEA (5 ng/ml), CA199 (37 ng/ml), Age (55 years) and tumor size (2.6 cm) were also obtained by ROC curve analysis, and they were classified.

### Statistical Analyses

If the quantitative data did not conform to the normal distribution, it was expressed as the median, and the difference was compared using the Mann-Whitney U test. The classified variables were represented by Numbers (%), and chi-square test was used for comparison of differences. The survival of the patients was analyzed by Kaplan-Meier method. COX regression model was used to analyze the risk factors affecting the prognosis of patients. To compare the predictive power of COUNT with NLR and PNI in the survival model, we performed additional implementations of areas under the curves (AUC), the net reclassification index (NRI) and integrated discriminatory index (IDI).IBM SPSS statistical software package V. 24.0 and R version 4.0 was used for statistical analysis, and P < 0.05 was considered statistically significant.

## Results

A total of 94 eligible patients were enrolled (63 men (67.0%) and 31 women (33.0%); age range, 31–73 years; mean age, 54.5 ± 8.6 years). The surgical methods varied according to different Bismuth-Corlette classification and intraoperative conditions, including 11 (11.7%) patients were staged as type I (11 cases of simple hilar resection), 9 (9.6%) patients as type II (7 cases of simple hilar resection, and 2 cases of square lobectomy), 18 (19.1%) patients as type IIIa (right hemihepatectomy in 6 cases, right hemihepatectomy combined with caudate lobectomy in 11 cases and square lobectomy in 1 case), 22 (23.4%) patients as type IIIb (8 cases of left hemihepatectomy, 3 cases of enlarged left hemihepatectomy, 11 cases of left hemihepatectomy combined with caudate lobectomy) and 34 (36.2%) patients as type IV (left hemihepatectomy 6, enlarged left hemihepatectomy 4, right hemihepatectomy 7, enlarged right hemihepatectomy 3, left hemihepatectomy combined with caudate lobotomy 12, enlarged right hemihepatectomy combined with caudate lobotomy 2). Among them, 29 tumors invaded the large blood vessels (portal vein and hepatic artery). We performed vascular reconstruction for 6 patients with portal vein invasion, including 2 cases with end-to-end anastomosis, 4 cases with partial sidewall resection of portal vein and vessel wall repair. In all patients, 42 (44.7%) had poorly differentiated, 30 (31.9%) had moderately differentiated and 22 (23.4%) had highly differentiated tumors. In order to analyze the relationship between preoperative CONUT score and clinicopathological characteristics of HCCA patients. We classify related variables according to their own characteristics or optimal cutoff values. As shown in **Table 3**, preoperative CONUT score were not associated with gender, age, preoperative TBIL, tumor size, histopathological type, Bismuth-Corlette classification, Hepatic parenchymal invasion, Lymph node metastasis, Hepatic artery invasion, Portal vein invasion, Perineural invasion and AJCC stage, but were associated with BMI (χ2 = 6.788, p=0.034), Degree of cure (χ2 = 8.840, p=0.003), Clavien-Dindo classification (χ2 = 7.144, p=0.008) and Bile leakage (χ2 = 6.546, p=0.011). Meanwhile, CONUT score was correlated with ALT (U=667.5, p=0.007), AST (U=720.0, p=0.022), ALP (U=713.0, p=0.034) OS: AUC = 0.717 (95% CI [0.615–0.820]) RFS: AUC = 0.722 (95% CI [0.621–0.823] (**Figures 4A** and **4B**) in clinical blood biochemical indicators, as shown in **Tables 2** and **3**.

**Table 2:** Baseline characteristics of patients with HCCA according to CONUT levels.

Variables	CONUT<3	CONUT≥3	U value	*P* value
Neutrophil (10^9^/L)	5.0(3.2–6.7)	5.3(4.2–7.7)	803.0	0.163
PLT (10^9^/L)	211(167–252)	226(185–262)	826.0	0.226
ALT (U/L)	42.3(22.3–124.4)	119(57.1–329.0)	667.5	0.007*
AST (U/L)	48.7(21.7–92.8)	91.1(31.7–222.3)	720.0	0.022
GGT (U/L)	199.(115.9–486.4)	267.(140.0–701.9)	864.0	0.294
ALP (U/L)	257.(133.6–363.3)	373.(214.3–480.7)	713.0	0.034*
PT (s)	12.1(11.3–12.4)	11.8(11.3–12.3)	871.5	0.398
APTT (s)	32.4(31.0–35.7)	33.6(30.9–36.0)	895.5	0.515
Operation time (min)	270(205–320)	245(220–315)	913.5	0.612

*Indicates P<0.05.

HCCA, Hilar cholangiocarcinoma; PLT, platelet; ALT, alanine aminotransferase; AST, aspartate aminotransferase; GGT, transaminaseγ-glutamyl transpeptidase; ALP, alkaline phosphatase; PT, prothrombin time; APTT, activated partial thromboplastin time.

**Table 3:** Relationships between CONUT score and clinicopathological characteristics of 94 HCCA patients

Variable	CONUT^low^ (n = 63)	CONUT^high^ (n = 31)	χ^2^value	*P* value
Gender			1.556	0.212
Male	30 (47.6%)	19 (61.3%)		
Female	33 (52.4%)	12 (38.7%)		
Age (years)			0.057	0.812
<55	26 (41.3%)	12 (38.7%)		
≥55	37 (58.7%)	19 (61.3%)		
BMI (kg/m^2^)			6.788	0.034*
<18.5	8 (12.7%)	11 (35.5%)		
≥18.5, <25.0	38 (60.3%)	13 (41.9%)		
≥25.0	17 (27.0%)	7 (22.6%)		
Preoperative TBIL (mg/dl)			0.928	0.335
<65.9	18 (28.6%)	6 (19.4%)		
≥65.9	45 (71.4%)	25 (80.6%)		
Tumor size (cm)			0.714	0.398
<2.6	26 (41.3%)	10 (32.3%)		
≥2.6	37 (58.7%)	21 (67.7%)		
Histopathological type			0.004	0.948
Poorly differentiated	28 (44.4%)	14 (45.2%)		
medium-high differentiation	35 (55.6%)	17 (54.8%)		
Bismuth-Corlette classification			1.661	0.197
I-II	11 (17.5%)	9 (29.0%)		
III-IV	52 (82.5%)	22 (71.0%)		
Hepatic parenchymal invasion			0.212	0.645
Positive	17 (27.0%)	7 (22.6%)		
Negative	46 (73.0%)	24 (77.4%)		
Lymph node metastasis			1.992	0.158
Positive	21(33.3%)	15 (48.4%)		
Negative	42 (66.7%)	16 (51.6%)		
Hepatic artery invasion			0.178	0.673
Positive	10 (15.9%)	6 (19.4%)		
Negative	53 (84.1%)	25(80.6%)		
Portal vein invasion			1.194	0.275
Positive	12 (19.0%)	9 (29.0%)		
Negative	51 (81.0%)	22 (71.0%)		
Perineural invasion			0.008	0.928
Positive	25 (39.7%)	12 (38.7%)		
Negative	38 (60.3%)	19 (61.3%)		
Degree of cure			8.940	0.003*
R0	61 (96.8%)	2 (3.2%)		
R1	23 (74.2%)	8 (25.8%)		
AJCC stage				
I-II	32 (50.8%)	10 (32.3%)	2.888	0.089
III-IV	31 (49.2%)	21 (67.7%)		
Clavien-Dindo classification				
<IIIa	54 (85.7%)	19 (61.3%)	7.144	0.008*
≥IIIa	9 (14.3%)	12 (38.7%)		
Bile leakage				
Presence	52 (82.5%)	18 (58.1%)	6.546	0.011*
Absence	11 (17.5%)	13 (41.9%)		

*Indicates P<0.05. HCCA, Hilar cholangiocarcinoma; BMI, body mass index; TBIL, total bilirubin; AJCC, American Joint Committee on Cancer; CONUT, controlling nutritional status; The cut off value of CONUT score is 3, according to the ROC analyses; R0 resection was confirmed to be negative for both proximal and distal bile duct resection margins by postoperative pathological examination. R1 resection showed no residual tumor in the naked eye, and postoperative pathological examination indicated that the cutting edge of the broken end was positive under the microscope.

**Figure 2:**
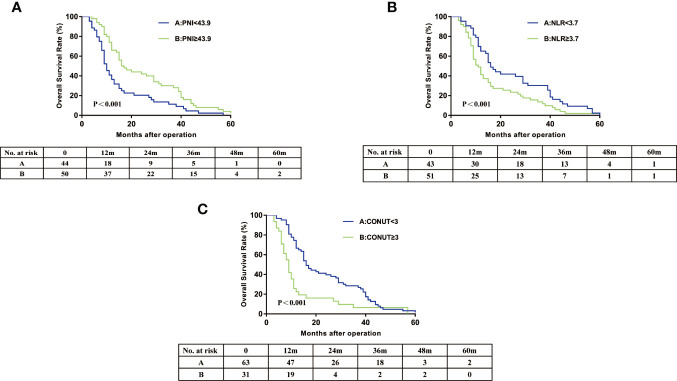
Overall survival curves for hilar cholangiocarcinoma patients according to PNI **(A)**, NLR **(B)** and CONUT score **(C)**. PNI, prognostic nutritional index; NLR, neutrophil lymphocyte ratio; CONUT, controlling nutritional status.

**Figure 3:**
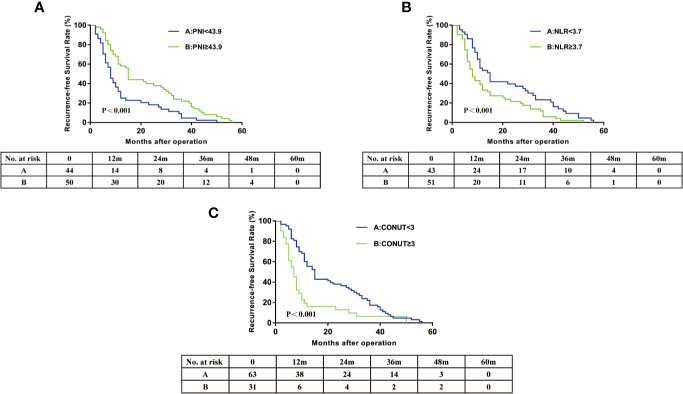
Recurrence-free survival curves for hilar cholangiocarcinoma patients according to PNI **(A)**, NLR **(B)** and CONUT score **(C)**. PNI, prognostic nutritional index; NLR, neutrophil lymphocyte ratio; CONUT, controlling nutritional status.

**Figure 4:**
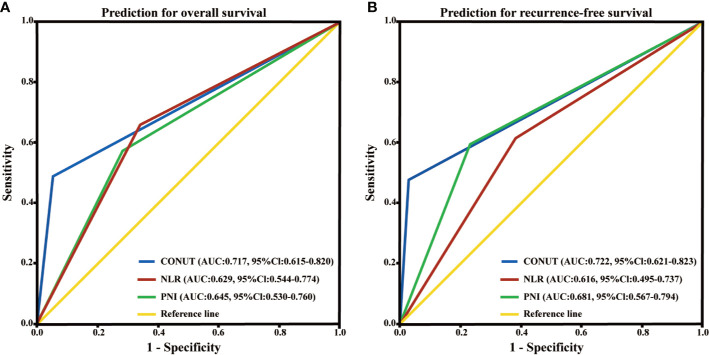
Time-dependent ROC curves of preoperative PNI, NLR and CONUT score for the prediction of hilar cholangiocarcinoma patients’ outcomes. **(A)** Overall survival. **(B)** Recurrence-free survival. PNI, prognostic nutritional index; NLR, neutrophil lymphocyte ratio; CONUT, controlling nutritional status; ROC, receiver operating characteristic; AUC, area under curve; CI, confidence interval.

In this cohort, the 3-year OS rates was 21.4% and 3-year RFS rates was 17.0%. The 3 year OS of the PNI≥43.9 group (24.0%) was significantly higher than that of the PNI<43.9 group (9.1%) (**Figure 2**). The 3 year OS rate of the NLR≥3.7 group (11.8%) was lower than that of the NLR<3.7 group (23.3%) (**Figure 2**). Patients with CONUT ≥3 had a significantly worse 3-year OS than patients with CONUT<3 (6.5 vs 22.3%) (**Figure 2**). The Kaplan-Meier OS curves show significant separation in each of the above groups. The Recurrence-free survival curves for PNI, NLR, and CONUT showed similar result (**Figures 3A–C**). We plotted the ROC curves of the three models and found that the AUC value of CONUT was greater than that of NLR and PNI (OS: AUC = 0.717 [95% CI (0.615–0.820)] RFS: AUC = 0.722 [95% CI (0.621–0.823); **Figures 4A, B**]. To compare their predictive power, additional NRI and IDI values were calculated, as shown in **Tables 4** and **5**. The NRI and IDI values (CONUT vs NLR) for Overall survival were 0.124 [95% CI (0.013–0.235), P = 0.009], and 0.009 [95% CI (0.005–0.013), P < 0.001]. Meanwhile, the NRI and IDI values (CONUT vs PNI) for Overall survival were 0.093 [95% CI (−0.022–0.208), P = 0.034], and 0.005 [95% CI (0.002–0.008), P = 0.004].

**Table 4:** Comparison of the predictive power of COUNT with NLR in the survival model.

	AUC (95%CI)	*P* value	NRI (95%CI)	*P* value	IDI (95%CI)	*P* value
Overall survival						
NLR	0.629 (0.544–0.774)	Ref.	Ref.	Ref.	Ref.	Ref.
CONUT	0.717 (0.615–0.820)	0.035*	0.124 (0.013–0.235)	0.009*	0.009 (0.005–0.013)	<0.001*
Recurrence-free survival						
NLR	0.616 (0.495–0.737)	Ref.	Ref.	Ref.	Ref.	Ref.
CONUT	0.722 (0.621–0.823)	0.007*	0.139 (0.024–0.254)	0.002*	0.015 (0.008–0.022)	<0.001*

*Indicates P<0.05. NLR, neutrophil lymphocyte ratio; CONUT, controlling nutritional status; NRI, net reclassification improvement; IDI, integrated discrimination improvement; AUC, areas under the curves; CI, confidence interval.

**Table 5:** Comparison of the predictive power of COUNT with PNI in the survival model.

	AUC (95%CI)	*P* value	NRI (95%CI)	*P* value	IDI (95%CI)	*P* value
Overall survival						
PNI	0.645 (0.530–0.760)	Ref.	Ref.	Ref.	Ref.	Ref.
CONUT	0.717 (0.615–0.820)	0.066	0.093(−0.022–0.208)	0.034*	0.005 (0.002–0.008)	0.004*
Recurrence-free survival						
PNI	0.681 (0.567–0.794)	Ref.	Ref.	Ref.	Ref.	Ref.
CONUT	0.722 (0.621–0.823)	0.127	0.060(−0.034–0.154)	0.126	0.001 (0.000–0.002)	0.028*

*Indicates P<0.05. PNI, prognostic nutritional index; CONUT, controlling nutritional status; NRI, net reclassification improvement; IDI, integrated discrimination improvement; AUC, areas under the curves; CI, confidence interval.

Univariate analyses showed that serum CA199 levels (<37 vs. ≥37 ng/mL; P < 0.05), tumor size (<3.1 vs. ≥3.1 cm; P < 0.05), lymph node metastasis (positive vs. negative; P < 0.05), portal vein system invasion (positive vs. negative; P < 0.05), AJCC stage (I–II vs. III–IV; P < 0.001), Degree of cure (R0 vs. R1; P < 0.001), Preoperative PNI (<43.7 vs. ≥43.7; P < 0.001), Preoperative NLR (<3.6 vs. ≥3.6; P < 0.05), Preoperative CONUT score (Low vs. High; P < 0.001) were related to OS and RFS (**Table 6**). All factors with P<0.05 in the univariate analysis were included in the Cox regression model, which were BMI, Tumor size, Lymph node metastasis, Clavien-Dindo classification, Degree of cur, Preoperative PLR, Preoperative CONUT score respectively. Since CONUT, NLR and PNI are mutually interfering factors, three multi-factor Cox proportional models are established to weaken the collinearity problem. In each model, Degree of cure (P < 0.05), CONUT (P < 0.001), NLR (P < 0.05), and PNI (P < 0.001) were still strong and independent prognostic factors for OS and RFS (**Table 7**).

**Table 6:** Univariate analyses of factors associated with overall survival and recurrence-free survival of HCCA patients.

Variable	OS			RFS		
	HR	95%CI	*P* value	HR	95%CI	*P* value
Gender (Male vs. Female)	1.01	0.60–1.70	0.975	0.66	0.39–1.10	0.107
Age (<55 vs. ≥55 years)	1.43	0.81–2.50	0.214	0.94	0.59–1.63	0.981
BMI (<18.5 vs.≥18.5, <25.0 vs. ≥25.0 kg/m^2^)	0.47	0.24–0.92	0.012*	0.60	0.31–1.17	0.069
Preoperative TBIL(<65.9 vs. ≥65.9 mg/dl)	1.85	0.96–3.59	0.066	1.44	0.79–2.61	0.233
Preoperative CEA (<5 vs. ≥5 ng/ml)	1.29	0.73–2.29	0.374	1.30	0.75–2.26	0.348
Preoperative CA199 (<37 vs. ≥37 ng/ml)	2.02	1.06–3.84	0.032*	3.58	1.69–7.59	0.001*
Tumour size (<2.6 vs. ≥2.6 cm)	1.74	1.01–3.03	0.048*	1.32	0.79–2.20	0.298
Histopathological type (Poor vs. medium-high)	0.59	0.34–1.01	0.056	0.76	0.46–1.27	0.294
Bismuth-Corlette classification (I–II vs. III–IV)	0.67	0.36–1.26	0.214	0.78	0.47–1.76	0.912
Hepatic parenchymal invasion (Positive vs. Negative)	1.18	0.65–2.14	0.584	1.15	0.65–2.03	0.637
Lymph node metastasis (Positive vs. Negative)	0.35	0.20–0.60	<0.001*	0.45	0.27–0.75	0.002*
Hepatic artery invasion (Positive vs. Negative)	1.50	0.71–3.17	0.289	1.59	0.76–3.36	0.219
Portal vein invasion (Positive vs. Negative)	0.45	0.24–0.86	0.015*	0.34	0.20–0.61	<0.001*
Perineural invasion (Positive vs. Negative)	1.11	0.65–1.89	0.701	1.40	0.82–2.39	0.216
AJCC stage (I–II vs. III–IV)	3.01	1.68–5.40	<0.001*	2.56	1.48–4.41	0.001*
Clavien-Dindo classification (<IIIa vs. ≥IIIa)	1.79	1.00–3.20	0.049*	2.15	1.24–3.71	0.006*
Degree of cure (R_0_ vs. R_1_)	12.5	5.07–30.77	<0.001*	11.7	5.35–25.7	<0.001*
Bile leakage (Presence vs. Absence)	0.93	0.52–1.67	0.801	1.06	0.61–1.84	0.848
Preoperative PNI (<43.7 vs. ≥43.7)	0.36	0.21–0.61	<0.001*	0.35	0.21–0.59	<0.001*
Preoperative NLR (<3.6 vs. ≥3.6)	2.78	1.59–4.86	<0.001*	2.17	1.29–3.66	0.004*
Preoperative CONUT score (Low vs. High)	3.77	2.21–6.43	<0.001*	3.62	2.16–6.06	<0.001*

*Indicates P<0.05. HCCA, Hilar cholangiocarcinoma; AJCC, American Joint Committee on Cancer; HR, hazard ratio; CI, confidence interval; OS, overall survival; RFS, recurrence-free survival; BMI, body mass index; CEA, carcinoembryonic antigen; CA199; carbohydrate antigen 199; PNI, prognostic nutritional index; NLR, neutrophil lymphocyte ratio; CONUT, controlling nutritional status.

**Table 7:** Multivariate analyses of factors associated with overall survival and recurrence-free survival of HCCA patients

Variable	OS			DFS		
	HR	95%CI	*P* value	HR	95%CI	*P* value
Model 1						
BMI (<18.5 vs.≥18.5, <25.0 vs. ≥25.0 kg/m^2^)	1.68	1.01– 2.78	0.045*	1.89	1.16–3.06	0.010*
Preoperative CA199 (<37 vs. ≥37 ng/ml)	0.85	0.39–1.87	0.692	1.75	0.76–4.05	0.189
Tumour size (<2.6 vs. ≥2.6 cm)	2.40	1.22–4.82	0.011*	1.43	0.77–2.65	0.255
Lymph node metastasis (Positive vs. Negative)	0.30	0.13–0.66	0.003*	0.50	0.24–1.03	0.061
Portal vein invasion (Positive vs. Negative)	0.61	0.28–1.30	0.199	0.43	0.21–0.88	0.020*
AJCC stage (I–II vs. III–IV)	1.22	0.49–3.02	0.673	1.10	0.48–2.52	0.815
Clavien-Dindo classification (≥IIIa vs. ≥IIIa)	1.90	0.90–3.99	0.092	1.74	0.86–3.51	0.124
Degree of cure (R_0_ vs. R_1_)	7.13	2.46–20.6	<0.001*	6.74	2.60–17.5	<0.001*
Preoperative CONUT score (Low vs. High)	5.30	2.58–10.9	<0.001*	4.01	1.97–8.18	<0.001*
Model 2						
BMI (<18.5 vs.≥18.5, <25.0 vs. ≥25.0 kg/m^2^)	1.13	0.72–1.77	0.603	1.36	0.89–2.07	0.162
Preoperative CA199 (<37 vs. ≥37 ng/ml)	0.94	0.46–1.93	0.872	1.88	0.85–4.17	0.122
Tumour size (<2.6 vs. ≥2.6 cm)	1.64	0.86–3.09	0.131	1.21	0.66–2.21	0.544
Lymph node metastasis (Positive vs. Negative)	0.41	0.19 –0.89	0.024	0.62	0.31–1.26	0.184
Portal vein invasion (Positive vs. Negative)	0.70	0.34–1.47	0.351	0.46	0.23–0.93	0.029
AJCC stage (I–II vs. III–IV)	1.24	0.53–2.92	0.625	1.11	0.51–2.44	0.796
Clavien-Dindo classification (≥IIIa vs. ≥IIIa)	3.25	1.66–6.39	0.001*	3.05	1.63–5.74	0.001*
Degree of cure (R_0_ vs. R_1_)	8.82	3.18–24.5	<0.001*	9.76	3.87–24.6	<0.001*
Preoperative PLR (≥3.6 vs. ≥3.6)	2.27	1.22 –4.24	0.010*	1.83	1.03–3.24	0.038*
Model 3						
BMI (<18.5 vs.≥18.5, <25.0 vs. ≥25.0 kg/m^2^)	1.15	0.74–1.79	0.546	1.36	0.89–2.07	0.151
Preoperative CA199 (<37 vs. ≥37 ng/ml)	0.93	0.43–2.03	0.85	1.79	0.77–4.15	0.175
Tumour size (<2.6 vs. ≥2.6 cm)	2.50	1.30–4.84	0.006*	1.57	0.85–2.89	0.150
Lymph node metastasis (Positive vs. Negative)	0.34	0.15–0.77	0.010*	0.53	0.25–1.12	0.097
Portal vein invasion (Positive vs. Negative)	0.56	0.26–1.20	0.134	0.41	0.20–0.83	0.013
AJCC stage (I–II vs. III–IV)	1.48	0.60–3.66	0.394	1.24	0.55–2.83	0.606
Clavien-Dindo classification (≥IIIa vs. ≥IIIa)	3.11	1.55–6.26	0.001*	2.65	1.40–5.05	0.003*
Degree of cure (R_0_ vs. R_1_)	5.10	1.78–14.6	0.002*	5.77	2.19–15.2	<0.001*
Preoperative PNI (≥43.7 vs. ≥43.7)	0.26	0.14–0.49	<0.001*	0.33	0.19–0.59	<0.001*

*Indicates P<0.05. HCCA, Hilar cholangiocarcinoma; AJCC, American Joint Committee on Cancer; HR, hazard ratio; CI, confidence interval; OS, overall survival; DFS, disease-free survival; BMI, body mass index; CA199; carbohydrate antigen 199; PNI, prognostic nutritional index; NLR, neutrophil lymphocyte ratio; CONUT, controlling nutritional status.

## Discussion

The influence of inflammation and immunonutrition on the prognosis of cancer patients is the basis of many studies, and there is an important relationship between malnutrition and increased inflammation and the poor prognosis of cancer patients (34, 35). Currently, tools to objectively assess nutritional status are poorly studied in cancer patients, while PNI, NLR, and CONUT scores can predict the prognosis of cancer patients, while they are objective and readily available (25, 36). Controlling nutritional status (CONUT) score is a relatively new immuno-nutritional biomarker, which has the advantages of simplicity, low cost and comprehensibility. Derived from total cholesterol, total lymphocyte count and serum albumin, it can assist in the assessment of nutritional status during hospitalization (37, 38). In two studies of patients with hilar cholangiocarcinoma after intrahepatic cholangiocarcinoma and adjuvant therapy, a high CONUT score was identified as an independent predictor of poor OS prognosis (19, 30). Similar to previous reports, univariate analysis in our study showed that preoperative PNI, NLR, and CONUT were associated with postoperative survival in HCCA patients.

Albumin is produced by the liver and is the most abundant protein in plasma. Serum albumin is an important factor in assessing the nutritional status of patients and has been widely reported to be closely related to the prognosis of various cancers, including HCCA (39–41). The expression of lymphocytes is critical in tumor defense and can promote the death of cytotoxic cells. Therefore, impaired tumor immune function may be associated with reduced number of such cells, leading to tumor progression (42–44). At the same time, neutrophil to lymphocyte ratio (NLR) has been reported as an independent prognostic factor in advanced patients receiving adjuvant therapy and patients undergoing surgery for biliary tract cancer (45, 46). In addition, TLC is involved in acquired and adaptive immunity, including humoral immunity against tumors, and is an important factor for cells to perform their immune functions. In summary, CONUT score can reflect not only nutritional status, but also immune status (47). Our research proves that the CONUT score, PNI, and NLR as immuno-nutritional indicators were independent prognostic factor for HCCA in both PFS and OS, and high preoperative CONUT scoring status was associated with shorter PFS and OS. In summary, our study is the first to demonstrate the prognostic significance of the CONUT score in patients after HCCA surgery.

Although the AUC value of the CONUT score in the ROC survival curve is only 0.72, it may be influenced by the small number of cases and the location, and its predictive effect is only average. However, in the survival model, the AUC value of CONUT score was higher than that of NLR and PNI. In order to further compare the predictive power between them, NRI and IDI values were calculated, and the results showed that the CONUT predictive model was more valuable than PNI and NLR models for OS and RFS of patients after HCCA. Total cholesterol has been reported to be associated with multiple tumor progression and patient survival (48–50). The CONUT score also took into account a certain levels of serum cholesterol compared with NLR and PNI. Therefore, we consider the results of the above study to be related to it.

Severe postoperative complications can prolong hospital stay, increase morbidity and worsen prognosis. The main complications after hepatectomy were pulmonary complications, refractory ascites, biliary leakage, abdominal hemorrhage, liver failure, etc. Recently, more and more studies have focused on the nutritional status of patients, and found that postoperative complications III-V are not only related to liver function reserve function, but also related to nutritional status. Nutritional intervention can improve patients’ tolerance to chemotherapy and surgery, reduce postoperative complications and improve prognosis (51–53). It was reported in relevant literature that higher CONUT score was associated with higher incidence of postoperative pneumonia, length of hospital stay, biliary leakage, pancreatic leakage and serious complications (14, 25, 54, 55). At the same time, the same results were also obtained in our study. High CONUT score was closely related to the occurrence of postoperative complications (Clavien-Dindo≥IIIa) and the occurrence of biliary leakage, and the reasons were considered to be related to the preoperative nutritional immune status of the patients. In addition, the degree of radical resection by “radical-intent resection” is related to CONUT, but it is greatly affected by confounding factors such as surgical mode.

Our study had some limitations. First of all, patients were grouped by the optimal cutoff values of CONUT, PNI, NLR, and other indicators. However, their critical values varied in different reports, and the optimal critical values were still unclear. Second, this was a retrospective study with some bias. Third, the number of patients included in this study is small, and further large-scale, prospective trials are needed to confirm our results.

## Conclusion

To sum up, our study is the first to demonstrate that preoperative CONUT score is an independent prognostic factor for postoperative survival of patients with HCCA. Meanwhile, the CONUT predictive model was more valuable than PNI and NLR models for OS and RFS of patients after HCCA.

## Data Availability Statement

The raw data supporting the conclusions of this article will be made available by the authors, without undue reservation.

## Ethics Statement

The studies involving human participants were reviewed and approved by the Ethics Committee of the Affiliated Hospital of Southwest Medical University. The patients/participants provided their written informed consent to participate in this study.

## Author Contributions

AW, ZH, and PC wrote the manuscript and performed data analyses. AW, BS, and HC collected the clinical data. YQ, TH, and YC reviewed the manuscript. WF and YP made key modifications to the manuscript and approved the final submission. All authors contributed to the article and approved the submitted version.

## Funding

Southwest Medical University Science and Technology Strategic Cooperation Applied Basic Research Project(2018LZXNYD-ZK14); Cooperative Science and Technology Project of North Sichuan Medical College (18SXHZ0435).

## Conflict of Interest

The authors declare that the research was conducted in the absence of any commercial or financial relationships that could be construed as a potential conflict of interest.
